# Dentistry in Foreign Countries

**Published:** 1898-03

**Authors:** 


### Abstracts and Translations.

### DENTISTRY IN FOREIGN COUNTRIES.

APPLICATION having been made to the Department of State for information as to the conditions governing the practice of dentistry in the various countries of Europe, and in the Hawaiian Islands especially, and the qualifications necessary for an American dentist, the diplomatic officers at the European capitals and the minister at Honolulu were requested to obtain the desired data. Their answers, in substance, are printed here for the general information of the dental profession of the United States. A note from the Mexican minister at Washington, in response to an inquiry from another source, as to the conditions in Mexico, is included in the series.

AUSTRIA-HUNGARY.—Replying to despatch of June 30 last, asking for information in regard to foreign dentists practising their profession in this country, I beg to reply that foreign dentists are practically excluded from practising in Austria; the same general law which governs the practice of medicine applies to dentistry. Every medical doctor or dentist must have passed the regular examinations of the various Austrian state preparatory schools, university, and medical colleges; no foreign diplomas of any kind are accepted as evidence of fitness in either profession. By this law any regular graduate of medicine may, if he prefers, practise dentistry without any further examination in that special branch of the profession. So far as I am able to learn, there is only one so-called American dentist practising his profession in this country, and he received a special permit to do so from the emperor, after having renounced his American citizenship and become a naturalized citizen of Austria. This special imperial permission allowed him to employ two assistants, without regard to their nationality.

LAWRENCE TOWNSEND,
*Secretary of Legation.*

VIENNA, July 29, 1896.

BELGIUM.—Foreign dentists are admitted to practise their profession in Belgium, provided they comply with the conditions prescribed in a royal decree of January 6, 1885.

All candidates, foreign or native, are required to pass an examination prescribed by the medical commission of the province in which they have resided during the two years that all such candidates must practise in the office of a regularly licensed dentist. This examination is announced in the *Moniteur Belge* a month before it is held. All candidates presenting themselves for this examination must be provided with a certificate showing that they have performed the preliminary two years' practice above mentioned. However, the Minister of the Interior and of Public Instruction may excuse from the production of this certificate any foreign candidate who has acquired the right to practise his profession in his own country.

JAS. S. EWING,
*Minister.*

BRUSSELS, August 25, 1896.

DENMARK.—I have met with much difficulty in obtaining the information desired as to practice of dentistry in Denmark by foreigners, for the reason that the ordinances and regulations are contained in several pamphlets, which are out of print and not on sale. But at length I have found copies and obtained leave to have them translated.

The general result is that dentists cannot practise in Denmark without a license, and such license cannot be obtained without passing an examination and a certain amount of service as assistant; and the applicant is not entitled to such examination without certain educational certificates and proofs of good character.

JOHN E. RISLEY,
*Minister.*

COPENHAGEN, September 11, 1896.

FRANCE.—In reply to department instruction of June 30, asking under what conditions foreign dentists are admitted to practise their profession in France, and whether the conditions prescribe an examination or not, I have the honor to furnish the following information:

According to Article 2 of the law of November 30, 1892, no one can exercise the profession of dentist in France if he is not provided with a French diploma of doctor of medicine or of surgeon dentist, issued by a superior medical institution of the state, after a certain course of studies fixed by the government and followed by public examination.

HENRY VIGNAUD,
*Secretary of Embassy.*

PARIS, July 16, 1896.

GERMANY.—On the receipt of the instruction of June 30 last, I addressed a note to the imperial foreign office, requesting, as directed, to be furnished with information as to the conditions under which foreign dentists are admitted to practise their profession in Germany. A reply to this note has now been received, and I have the honor to transmit herewith a copy, with translation, of the same.

EDWIN F. UHL,
*Ambassador.*

BERLIN, October 2, 1896.

[Translation.]

FOREIGN OFFICE, BERLIN, October 1, 1896.

Referring to the communication of July 18 last (F. O., No. 69), the undersigned has the honor to inform his excellency the ambassador extraordinary and plenipotentiary of the United States of America, Mr. Edwin F. Uhl, that no official permit is necessary for practising dentistry in Germany. Those persons, however, who wish to use the title of "doctor of dentistry," or another title of equal value, require, according to section 29 of the trade regulation (as adopted July 1, 1883, imperial law sheet, p. 177ff), a permit (approbation), which, with certain exceptions prescribed by the chancellor of the (North German) union in his proclamation of December 9, 1869 (law sheet, p. 687), can only be granted after passing an examination according to the regulations contained in the publication of the imperial chancellor of July 5, 1889 (central sheet for the German empire, p. 417).

The regulations referred to, as well as another publication referring to this subject (central sheet for the German empire, 1890, p. 81), are herewith inclosed.

HOLSTEIN.

GREECE.—In accordance with the instruction of the 30th ultimo, I have the honor to inclose herewith a statement of the conditions under which foreign dentists are admitted to practice in Greece. As soon as possible, I shall forward the regulations on the subject in force in Roumania and Servia.

All persons desiring to obtain permission to practise dentistry in Greece must submit an official certificate from a recognized dental school or from a recognized dentist, certifying that they have been instructed and are proficient in dentistry.

Their examination shall be conducted either in the Greek language or in a foreign language. The subjects of examination are: (1) anatomy of the mouth and of the jaw-bones, (2) physiology of the teeth, (3) pathology and therapeutics of the teeth, (4) operations on the teeth, and (5) mechanical dentistry. The examination fee is twenty drachmas (about two dollars and thirty cents), and the fee for a certificate of permission to practise is ten drachmas (about one dollar and fifteen cents).

Foreign diplomas are authenticated on payment of four hundred drachmas (about forty-six dollars) for the stamped paper required. Without previous authentication of their diplomas, applicants from foreign countries are not allowed to present themselves for examination. It will be seen from the foregoing that all applicants, whether Greeks or foreigners, are required to pass an examination before permission to practise is granted, the only distinction made between Greeks and foreigners being that the diplomas of foreigners must first be verified.

E. ALEXANDER,
*Minister.*

ATHENS, July 23, 1896.

HAWAIIAN ISLANDS.—In reply to the instruction of June 30, relative to the conditions under which foreign dentists are permitted to practise their profession in the Hawaiian Islands, I have the honor to inclose herewith a copy of chapter 72 of the session laws of 1892, which will furnish the desired information.

ELLIS MILLS,
*Chargé d'Affaires ad interim.*

HONOLULU, August 6, 1896.

Sections 1, 6, and 7 are alone given.

SECTION 1. From and after the passage of this act it shall be unlawful for any person or persons to practise dentistry in the Hawaiian kingdom except upon a certificate issued from a board of dental directors.

SEC. 6. From and after sixty days subsequent to the passage of this act, the said board shall issue a certificate of qualification to any person who shall present a diploma from a reputable dental college or who shall pass a creditable examination before the board.

SEC. 7. Any person or persons receiving certificates from said board shall present said certificates to the Minister of the Interior, who shall record the same in a book kept for such purpose.

ITALY.—I have the honor to refer to the department instruction of June 30 last, on the subject of the admission to practice in Italy of foreign dentists, and to inclose accordingly herewith the copy and translation of a note which has been received to-day from the foreign office in reply to inquiries in the matter, together with the pamphlet referred to therein.

I beg to add that as there is no regular school of dentistry in Italy, some little informality in practice has existed, at least locally in Rome, in permitting foreigners to follow their profession, and on presentation before the proper officials of satisfactory credentials the applicants have been told they might practise among the people of their own nationality. But lately there has been begun an agitation against the granting of even that limited privilege and for the strict enforcement of the laws, information concerning which is contained in the before mentioned note from the foreign office.

LARZ ANDERSON,
*Secretary of Embassy.*

ROME, August 4, 1896.

[Translation.]

MINISTRY OF FOREIGN AFFAIRS, August 3, 1896.

In consideration of this, foreign dentists, too, in order to practise in Italy, must be furnished with the diploma for physic and surgery, and, according to Article 33 of the above-mentioned law, must have complied with the provision of Article 140 of the law of the 13th of November, 1859, with regard to public education, and which reads as follows:

"Examinations passed and degrees obtained outside the kingdom will have no effect in the state, except by some special law. Nevertheless, those who shall have obtained degrees in any of the Italian universities, or in some foreign university of greater importance, and shall prove to have really undergone the studies and examinations required for similar degrees in the universities of the state, will be freed from the condition of passing the special examination, and will be admitted, without further difficulty, to the general examination for the degrees which they care to take."

MEXICO.—Under date of June 3, 1896, Mr. Romero, minister from Mexico to the United States, writes to the Secretary of State:

In reply to your letter of the 1st instant, I have the honor to state that in order to practise the profession of dentistry in Mexico, it is not necessary to previously undergo an examination, it being sufficient to have the proper diploma issued by a foreign dental college, and the certificate duly authenticated by the competent authority in consideration of which that college has the legal power to issue such diplomas. Neither do the Mexican laws require the interested party to pass an examination in the Spanish language.

This has been the practice followed, but in case there may recently have been made some change of which I am ignorant, I will address the government of Mexico on this point, and, if so, I will have the honor to inform you.

NETHERLANDS.—Referring to department instruction, dated June 30, 1896, relating to dentistry in the Netherlands, I have the honor to inclose herewith a copy of the laws of December 25, 1878, and of March 26, 1895, relating to the subject.

Articles 8 and 9 of the first-mentioned law, a translation of which is annexed, prescribe the examinations to which persons desiring to practise as dentists in the Netherlands shall submit, and Articles 3 and 5 of the latter law, a translation of which is also attached, specify the foreign certificates or diplomas which permit a candidate to appear before the Netherlands examining committee for examination. The foreign certificates or diplomas thus recognized are the Belgian, German, British, French, Austrian, and Swiss medical diplomas, and the Belgian, German, British, French, and Swiss dental diplomas, in addition to those issued in the Dutch Indies, Surinam, and Curaçoa.

WILLIAM E. QUINBY,
*Minister.*

THE HAGUE, September 2, 1896.

PORTUGAL.—I have the honor to acknowledge the receipt of instruction dated June 30 last, addressed to Mr. Wilbor, in charge, and in reply have to state that an examination in the Portuguese language before the lyceum is imperative for all persons who desire to practise medicine or surgery in any of its branches in the kingdom.

This examination is duly certified to. The candidate is closely examined before the school of medicine, in the Portuguese language; in the case of dentists, in the anatomy of the head. Both of these examinations are rigorous. Once successfully passed, the candidate, upon the payment of about sixty dollars, is entitled to practise.

I inclose herein a digest of the medical law of July 13, 1870, still in force, bearing upon this subject.

GEO. WM. CARUTH,

*Minister.*

LISBON, August 12, 1896.

RUSSIA.—Referring to the instruction of June 30, I have the honor to transmit herewith copy and translation of Mr. Chichkine's note of September 11–23, and his accompanying inclosure of the law and regulations relating to the practice of dentistry within the empire for Russians and foreigners.

CLIFTON R. BRECKINRIDGE,
*Minister.*

ST. PETERSBURG, September 25, 1896.

[Translation of Mr. Chichkine's note.]

IMPERIAL MINISTRY OF FOREIGN AFFAIRS,
DEPARTMENT OF INTERIOR RELATIONS,
ST. PETERSBURG, September 11–23, 1896.

MR. MINISTER,—In reply to the note of June 29 (July 1) last, I have the honor to transmit herewith an extract of the laws of the empire containing the necessary prescriptions for Russian or foreign dentists who desire to acquire the right to practise in Russia.

I avail myself, etc.,
CHICHKINE.

MR. CLIFTON R. BRECKINRIDGE, ETC.

[Translation.]

ARTICLE 93. No person, either Russian or foreign, who does not possess a diploma or certificate from the universities and Military Medical Academy is allowed to practise any branch of medicine or to practise as a veterinary in Russia.

*Supplement to Article 596, Section Third.*—(*Section 32.*) Persons desiring the title of surgical dentist must have finished with success the studies at the medical-dentistry school and must undergo an examination by the examining committee of the university or Military Medical Academy.

*Section 31.* Persons desiring the title of dentist must present (1) a certificate, attested to by the local medical board, to the effect that the petitioners have studied dentistry for a period of three years with some well-known dentist, and that they have performed various dental operations on living beings with the proper skill and knowledge; (2) they must undergo an examination on the formation of the human skull, teeth, and gums, on the diseases the same are liable to, and on the means of curing by local means, being such as dentists are authorized to employ; (3) they must also undergo practical examinations at the hospitals, and perform several dental operations on dead bodies and living beings.

SPAIN.—Under date of September 28, 1896, the Spanish minister at Washington replies to an inquiry from the Department of State as follows:

In reply to your esteemed favor of the 26th of June, inquiring under what conditions American dentists are admitted to practice their profession in Spain, I take pleasure in inclosing a copy of the decree regulating the matter, which was recently forwarded to me from the proper department at Madrid.

You will no doubt notice that the contents of said decree refer chiefly to the medical profession, but dentists are admitted to practice under the same conditions as medical doctors.

The decree in question, dated February 6, 1869, provides that foreigners may enter the universities and other educational institutions of Spain on the same terms with Spaniards upon submitting to the rules in force. Persons who may have obtained academical degrees abroad may be admitted to the same examinations as those to which Spaniards are subjected, provided the authenticity of the degrees is established. The fees are the same as those paid by Spaniards. After having received a Spanish degree, foreign physicians must submit to the regulations decreed for Spanish physicians. Article 6 says, " To practise the medical profession, it shall suffice to present the degree acquired in a foreign public institution and to pay two hundred escudos on obtaining the authorization, which shall be given on receipt of the certified degrees." Article 8 provides that the foreign degree must be one that is recognized as valid in Spain. Application must be made on stamped paper, addressed to His Excellency the Minister of Public Works, accompanied by the original degree and a translation of it into Spanish made by the interpreter at the Department of Foreign Affairs.

SWEDEN.—The surgeon-general informs me that Swedish citizenship is required for every one wishing to practise a profession in Sweden, but there is nothing to prevent surgeon dentists wishing to practise under the rules imposed upon Swedish dentists, addressing a supplication to the king, stating their intention of becoming Swedish citizens. Applications must always be accompanied by certificates, and the applicants must pass the regular examination.

The surgeon-general informs me that there have been many such applications presented, but he could not recall a single instance of permission having been granted.

There has been, as yet, no compilation of the laws relating to dentistry in Norway, but I understand that one is in the course of preparation, and if I can secure a copy will forward it later.

T. B. FERGUSON,
*Minister.*

STOCKHOLM, October 1, 1896.

SWITZERLAND.—I have the honor to acknowledge the receipt of the instruction of June 30, 1896, asking under what conditions foreign dentists are admitted to practise their profession in Switzerland, and whether these conditions require an examination or not. In answer to this inquiry, I have the honor to inclose herewith a copy, in the German language, of a paragraph of a communication recently received at this legation from Professor Dr. Courvoisier, president of the Federal Medical Commission of Switzerland. I also transmit to the department by separate mail five small pamphlets of printed matter,—one in German, the others in the French language,—which, I am advised, embrace all the regulations now in force in Switzerland applicable to the practice of dentistry by foreigners. From these and other sources I understand (1) that no foreign diploma is recognized in Switzerland; (2) that all foreigners desiring to practise dentistry must submit to a thorough practical examination and to the maturitats examination; (3) that all examinations must be conducted in the French, German, or Italian language.

The practical effect of these regulations is the exclusion of all, or nearly all, American dentists, as a residence of several years in Switzerland, or a regular course in one of their universities, would be necessary to qualify them to successfully pass an examination. In answer to the remonstrance raised by American dentists here against these regulations, the response is made that identically the same requirements are exacted of native practitioners.

JOHN L. PEAK,

*Minister.*

BERNE, August 6, 1896.

UNITED KINGDOM.—Referring to the instruction of the 30th of June last, in relation to the conditions under which foreign dentists are admitted to practice in Great Britain, I have the honor to inclose herewith a copy of a note addressed by Mr. Bayard to Lord Salisbury on the 15th ultimo, together with a copy of his lordship's reply of the 1st instant on this subject.

JAMES R. ROOSEVELT,
*Secretary of Embassy.*

LONDON, August 5, 1896.

MR. BAYARD TO THE MARQUIS OF SALISBURY.

EMBASSY OF THE UNITED STATES,
LONDON, July 15, 1896.

MY LORD,—Referring to your lordship's note of the 23d of July, 1889, relating to the condition under which foreign dentists are admitted to practice in the United Kingdom, I have the honor to inform your lordship that I am instructed by my government to ask whether the regulations referred to prescribe an examination or not, and if they have been in any way changed or added to since the date of the above-mentioned note.

I shall also be extremely obliged to your lordship if additional copies of all laws and regulations upon the subject can be kindly furnished me, for the use of my government. I have, etc.,

T. F. BAYARD.

The Most Honorable The MARQUIS OF SALISBURY, K.G., etc.

LORD SALISBURY TO MR. BAYARD.

FOREIGN OFFICE, August 1, 1896.

SIR,—With reference to Mr. Bayard's note of the 15th ultimo, making further inquiries as to the conditions under which foreign dentists are admitted to practice in the United Kingdom, I have the honor to state that the lord president of the council has been informed by the president of the General Medical Council, to whom the matter, being of a technical nature, was referred, that the provisions of sections 8, 9, and 10 of the dentists' act, 1878, which governs the registration of foreign (and colonial) dentists with recognized certificates, imposes on the General Medical Council the duty of deciding whether the certificate entitles the holder to practise dentistry in his own state (or colony), and whether such certificate furnishes "sufficient guaranty of the possession of the requisite knowledge and skill for the efficient practice of dentistry or dental surgery."

In accordance with this duty, the General Medical Council has decided to recognize only such certificates as are evidence of a course of study and examination in arts and in dental surgery not inferior to those of British dental practitioners.

As the result of an inquiry held by them in 1879 into the sufficiency of the degree granted by the various American dental licensing bodies who applied for recognition, the University of Harvard and the University of Michigan were recognized as bodies granting "qualifications" which entitled the holders to registration in the United Kingdom, and no other application was granted. But after investigation in 1893 by the education committee of the council, it was found that the curricula of these two bodies were not considered adequate to insure the possession of the necessary qualifications, and the council thereupon resolved that the recognition of the dental degrees of the two universities above mentioned should be suspended until further notice. Consequently, no foreign dental degrees are at present recognized.

I beg leave to inclose copies of the medical acts from 1858 to 1886 (including the dentists' act, 1878), of the report of the education committee referred to above, and a report of the same committee, which will show the course of study and examination required for British dentists.

I have, etc.,

SALISBURY.

